# One-Year Effects of Project EX in Spain: A Classroom-Based Smoking Prevention and Cessation Intervention Program

**DOI:** 10.1371/journal.pone.0130595

**Published:** 2015-06-19

**Authors:** María T. Gonzálvez, José P. Espada, Mireia Orgilés, Daniel Soto, Steve Sussman

**Affiliations:** 1 Department of Health Psychology, Miguel Hernandez University, Elche, Spain; 2 University of Southern California, Los Angeles, California, United States of America; University of Westminster, UNITED KINGDOM

## Abstract

**Background:**

Tobacco use prevalence rates are high among Spanish adolescents. Programming to counteract tobacco use is needed.

**Methods and Findings:**

The current study provides a one-year follow-up outcome evaluation of Project EX, an eight-session classroom-based curriculum. The intervention was tested using a randomized controlled trial with 1,546 Spanish students, involving three program and three control schools. Compared to the control condition, the program condition revealed a greater reduction in nicotine dependence (*p* < .05) and CO ppm levels (*p* < .001), and lower consumption of cigarettes at last month (*p* = .03).

**Conclusions:**

Long-term outcomes of the Project EX classroom-based program are promising for adolescent prevention and possibly cessation in Spain.

## Introduction

Tobacco use has become a pediatric epidemic in Spain, because most new smokers begin at school age [1], and most regular adolescent tobacco users continue to use tobacco into adulthood [2]. The latest survey from the European School Survey Project on Alcohol and Other Drugs found that 54% of young Europeans aged 15 years had smoked cigarettes at least once in their lifetime, and more than half of the lifetime smokers had smoked cigarettes in the last 30 days (approximately 30% of all youth) [3]. These data may be contrasted with that of the United States, where approximately 7% of high school students indicated some form of tobacco use at least once in the previous month [4]. In Spain, tobacco and alcohol are the most consumed drugs in adolescence, and prevention and control of tobacco use is one of Spain’s most pressing public health needs [5, 6]. Tobacco education may be most fruitful when provided before adulthood, in order to prevent initiation, avoid cumulative physical consequences, and financial and societal costs [2]. Relatively few studies of teen smoking cessation have been conducted worldwide compared to adult cessation programs, with 25% of these studies being conducted outside the United States [7]. Also, while some tobacco use related prevention programming has been evaluated in Spain [8], only Project EX has been well-evaluated on the potential for prevention and cessation effects among adolescents in Spain [9], and provides motivation-coping skills-commitment material [10]. However, only its immediate effects are known.

In its clinic version, Project EX has shown positive 3-to-6-month outcome effects over three experimental trials and one multiple baseline single group trial [11–14]. While the school-based clinic version of Project EX has been found to be effective in the U.S., China, and Russia, international translation research on Project EX thus far has been limited to those smokers who attend the clinic. The classroom version of Project EX sought to increase program reach and is delivered in the classroom setting, with both tobacco users and nonusers. A prevention/cessation classroom-based version of EX was delivered in southern California and showed effects across the full range of smoking, and most strongly on cessation [15].

This paper focuses on the one-year self-reported behavioral outcomes of Project EX prevention/cessation program with Spanish adolescents. A previous paper reported on the implementation and immediate outcomes of the project [9]. The program was well-received and produced immediate effects on reducing smoking intentions and CO ppm levels. We hypothesized that the classroom-based curriculum would show maintenance of effects among the full sample at one-year follow-up, which would bolster the practical importance of program effects in Spain, as variations in sustained use or cessation are observed at the follow-up [16].

## Materials and Methods

### School Selection and Experimental Design

Before proceeding with the recruitment of the sample, the study was approved by the IRB at Miguel Hernandez University. The education authorities were informed of the study goals, and authorization was requested. Once such authorization had been obtained, the researchers interviewed the head teachers and the school counselors, informing them verbally and in writing about the aim of the study, so as to obtain their permission and encourage their cooperation. Finally, parents were informed by letter and requested to provide written consent for their children to participate in the study. The written parental consent was provided for all minors participating.

We used a convenience sample of 45 schools from 17 towns in the Province of Alicante. After the first in-person meeting with the school board of each school to present the objectives of the intervention, a total of six high schools from three cities (Elche [n = 4], Crevillente [n = 1] and San Vicente [n = 1]), were recruited (recruitment rate = 13%). The reasons for non-recruitment were no response (72%), responded back to us after a first meeting with a statement of no interest (18%) and inability to be able to implement the study during the school day (10%). The schools recruited were randomly assigned to one of two experimental conditions: treatment or standard care (control); that is, there were three schools per condition (schools were carefully matched into pairs prior to assignment).

### Project EX Curriculum

The original version of the EX prevention/cessation curriculum was translated into Spanish by two translators. Two bilingual researchers working at the University Miguel Hernandez (UMH) checked the translation by reading both the English and Spanish versions. The feasibility of the Spanish version was assessed in one focus group, composed of 10 students at a high school before program implementation, which verified that the curriculum was clearly understood and culturally appropriate.

The Project EX classroom curriculum is closely adapted from the clinic program [17, 18]. The learning activities included strategies to not start or quit smoking and learning skills for cessation maintenance, with a motivation-enhancement, interactive protocol. The program is comprised of several novel activities including: 1) role playing (“talk shows”), where student volunteers fill the role of each guest and the audience (other students in class) direct questions to the guests; 2) alternative medicine strategies including healthy breathing, characterized by slow and deep breaths, and yoga and meditation activities, which involves focusing attention on the present moment; and 3) a game, “is smoking on the menu” which divides the participants into teams to answer questions about tobacco. The curriculum involves eight sessions delivered in classrooms over a six-week period. The first four sessions are conducted over in a two-week period. The second four sessions are held once per week over the course of the following month. The sessions and their objectives are shown in Table 1. A more detailed description of the sessions can be found in the previous paper on the implementation and immediate outcomes of the project [9].

**Table 1:** Project EX curriculum.

Session name	Contents
*Orientation*	Imparts the ground rules for the class and discusses reasons for using, not using, quitting tobacco, or remaining tobacco free
*Tobacco affects your life*	Discusses how tobacco use can cause, rather than relieve stress
*Health dangers of tobacco use*	Discusses the harmful substances in tobacco and how it can injure one's body
*Quitting step 1-Making a commitment about not using tobacco*	Discusses addiction to tobacco. Methods of quitting and physical and psychological aspects of withdrawal are discussed
*Quitting step 2-Managing withdrawal symptoms*	Discusses more about nicotine, addiction, and strategies of avoiding addiction or managing withdrawal symptoms. Psychological coping includes self-forgiveness and avoiding false expectations regarding how not using tobacco or quitting will and will not affect one's life
*Taking care of a healthy body*	Involves learning lifestyle balance strategies, including weight control and practicing a “yoga activity”
*Taking care of your piece of mind*	Involves learning more coping strategies, including assertiveness training and anger management. Participants also learn the “letting feelings pass” meditation activity
*Not smoking again*: *commitment and avoiding relapse*	Involves learning means to avoid using tobacco again, or staying tobacco free, and mentions how topics covered in the tobacco education program could be applicable to other substances

### Cultural Adaptations

In addition to language adaptations, five changes were made in the curriculum to adapt it to Spanish culture. First, the original curriculum targets “tobacco” use including smokeless tobacco, pipes, cigars, and chewing tobacco. Spanish adolescents very rarely use other forms of tobacco other than cigarettes [5, 6], so that information was removed and the program focuses on prevention or cessation of cigarette smoking only. Second, when Project EX was implemented as a research project in the United States, extrinsic motivators were provided as incentives. Participants were told that they would obtain credits for participating in the program. In Spain, as in other international settings within which Project EX is being translated [11, 7], there were no incentives for attendance at the program sessions. Third, all names of characters in the talk shows [18] were changed from American to Spanish names. Fourth, the monetary amounts were changed from dollars to euros. For instance, in Session 1, “Orientation,” the original curriculum provides as a suggestion for the question “What are some reasons for quitting?” that one may “Save money. Over US $2,008 per year is spent on smoking one pack per day.” The amount was changed to 1,249 euros, the cost in Spain. Finally, in Session 3, “Health Dangers of Tobacco Use,” there is a question list for a game in the original curriculum regarding second-hand smoke and policies. Questions and forced-choice responses were changed to reflect policies in Spain. For example, one question asks “In which of the following places is it legal to smoke in the United States? (‘Airline flight,’ ‘Interstate bus,’ ‘New York City taxi cab,’ and ‘None of the above’.)” This item was replaced with “In which of the following places is it legal to smoke in Spain? (‘Airport,’ ‘Bus station,’ ‘Taxis,’ and ‘None of the above’ [correct answer]).”

### Training in Project EX

Between October and November of 2012, psychology graduate students at Miguel Hernandez University were given the opportunity to become a program facilitator. All volunteers received an introductory lecture about Project EX, which included a brief summary of the Project EX curriculum, its history, advantages of using the program, and the role of program facilitators. Seventeen Spanish graduate students (16 female), an average of 23 years of age (*SD* = 0.8), interested in implementing the program, spent eight hours studying and practicing the eight sessions of Project EX, including learning details of program delivery and how to deliver the material with fidelity. The training was provided by a researcher who had previously been trained by the program developer. All persons trained delivered the program to an average of two classrooms each.

### Participants

An average of 12 classes was selected per school, with a range of 8 to 19 classes, across the six high schools. A total of 1,546 students were selected for participation in the study (716 in the control condition and 830 in the program condition), 57% of the total enrollment across these classes (*n* = 2,691). Among the 1,546 subjects that participated in the pretest survey, 965 (393 in the control condition and 572 in the program condition) also completed one-year follow-up questionnaires (62.4% retention rate). All adolescents who conducted assessments at baseline and 12 months follow-up were included in the analysis.

Participants varied from 14 to 20 years of age (*M* = 15.03; *SD* = 1.01). The sample was 50.7% male; 91.8% were Spanish and 8.2% were other nationality (e.g., South America, Morocco, Eastern Europe). Further, 80.9% of the students lived with both parents and 36.1% of youths’ fathers and 36.6% of youths’ mothers completed high school. Approximately 32% of the participants smoked a cigarette sometime in their lives, 13.4% smoked in the last 30 days, and 7.9% smoked on the day of the pretest assessment.

### Data Collection and Measures

Pretest and one-year follow-up measures were collected from students using a self-report, closed-ended and fill-in-the-blank response questionnaire administered over one class period. At the one-year follow-up, students who failed to return the absentee survey were contacted by telephone for survey administration. Demographic items included age (in years), gender, nationality (born in Spain, or immigrated to Spain from another country), current living situation (with parents, live alone, other situation), and parents' education (mean response across father's [or stepfather's] and mother's [or stepmother's] educational levels) [19].

Smoking behavior was assessed with the fill-in-the blank item asking “How many cigarettes have you smoked in the last month (30 days)?” The level of nicotine dependence was assessed with the 8-item modified Fagerstrom Tolerance Questionnaire (mFTQ) [11, 20, 21]. The total score is the sum of scores for the items ranging from 0 to 25. To assess smoking intention [22], students were assessed with the question “How likely is it that you will smoke cigarettes in the next 12 months?” with response categories in a Likert scale format (1: Definitely not, 2: Probably not, 3: A little likely, 4: Somewhat likely, and 5: Very likely). Furthermore, to validate assessment-day smoking responses, assessment of expired CO was completed at pretest and one-year follow-up by use of a breath CO monitor (Micro+ Smokerlyzer; Belfont Technical Instruments, Kent, UK; http://www.bedfont.com/ch/smokerlyzer/micro, accessed 4-19-2014). Participants who responded to our one-year follow-up survey (15.75% of the total sample at one-year follow-up) did not complete the CO readings.

### Data Analysis

To assess the potential sampling bias due to attrition at the one-year follow-up across conditions (external invalidity), we calculated a new variable called “attrition group” in which we included 965 participants that were surveyed at both time points and compared them with the 581 participants that were only surveyed at pretest. We used logistic regression analysis with “attrition group” as the dependent variable. Predictors included age, gender, nationality (born in Spain, or not), current living situation, parents' education, assessment-last month smoking, level of nicotine dependence, and smoking intention. To assess the potential sampling bias due to attrition at the one-year follow-up as a function of condition (internal invalidity), comparisons were made between the sample that was lost at one-year follow-up and the rest of the sample that remained in the study at one-year follow-up, as a function of condition. The comparisons utilized were chi-square or t-test models to indicate statistically significant differences (two-tailed *p* value at the .05 level). Again, all relevant demographic and outcome variables were examined.

Data analysis for four outcomes variables, which compared the two conditions, was completed with multi-level analysis [23] by using IBM SPSS Statistics 21 [24]. The outcome variables evaluated in this analysis were smoking intention, last month smoking behavior, and nicotine dependence (using mFTQ score). We also examined CO readings. Condition was considered a fixed effect variable; fixed at desired experimental levels (school, considered as a random effect variable). The variables adjusted for in the analyses included baseline measurements for each respective outcome variable: age, gender, status of living with both parents, and attrition propensity. The propensity for attrition score was calculated from a model predicting the actual attrition status with pretest measures [25]. The pretest measures in the attrition propensity prediction models included age, gender, ethnicity, whether or not participant lived with both parents, program condition, and last month cigarette use. Two-tailed significance tests were employed for the significance level calculation in all analyses. In an additional analysis, intent-to-treat (ITT) quit rates were calculated for those who said that they had smoked in the last 30-days on assessment day at baseline.

## Results

### Assessment of Attrition Bias at Follow-up

First, regarding the analysis of external invalidity, differences were only found on age, gender, and school (see Table 2). Retained youth were slightly younger and a greater percentage female. The attrition analysis comparing conditions (internal invalidity) revealed statistically significant differences in nationality, smoking intention, and nicotine dependence between those who were retained at follow-up. The participants that were followed-up at one-year follow-up were in a higher percentage of Spanish nationality in the control condition compared to the program condition (93.9% versus 90.6%, respectively; (χ2(1) = 14.55; *p* < .05). In addition, those followed-up in the program condition were more likely to intend to not smoke in the future than in the control condition (61.1% versus 38.9%; *t*(1,539) = 6.87; *p* < .05), and had less nicotine dependence than in the control condition (58.5% versus 41.5%; *t*(1,391) = 23.67; *p* = .001). Differences in these variables have been controlled for in the analysis.

**Table 2:** Comparisons of key variables between lost-to-follow-up sample and subjects retained at one-year follow-up.

		Lost		Retained		*p*	*β*	*OR*
		Mean	SE	Mean	SE			
Age (years)		15.69	1.34	15.03	1.02	<.0001	0.36	1.44
Gender (% male)		58.60	2.30	50.60	1.80	.004	0.36	1.44
Nationality (% Spanish)		88.70	0.70	91.90	0.60	.90	-0.09	0.90
Live with… (%)	Both parents	39.00	2.40	61.00	1.90	.11	-0.29	0.74
	Mother	47.00	1.23	24.00	0.87	.80	-0.16	0.68
	Father	14.00	2.60	15.00	1.65	.80	-0.25	0.72
Parents education		27.00	2.20	48.50	3.15	.16	0.49	1.64
School	Cayetano Sempere	7.00	1.10	8.50	0.90	.01	0.65	1.92
	Sixto Marco	20.30	2.10	25.00	1.70	.72	0.064	1.06
	Tirant lo Blanc	19.10	2.60	24.90	2.10	.56	1.21	1.36
	San Vicente	17.40	10.10	3.30	8.10	<.0001	2.71	1.50
	Canónigo Manchón	29.70	4.00	20.40	3.20	<.0001	0.87	1.39
	La Asunción	6.50	6.60	17.90	5.30	.34	1.65	1.59
Cigarette smoking	30 day	0.83	3.02	0.75	2.46	.06	-0.70	0.93
	Smoking intention	24.00	1.90	76.00	1.10	.26	0.09	1.10
	FTQ Nicotine Dependence	24.50	1.10	75.50	0.60	.09	0.20	1.22

Note: The simple effects were analyzed with t-test or chi-square for each variable

### Effects on Smoking Intention and Tobacco Use

As shown in Table 3, the program was found to be statistically significant in reducing nicotine dependence and number of cigarettes at last month. Subjects in the control condition reported a higher level of nicotine dependence at the one-year follow-up (*p* < .05) and increased consumption of cigarettes (*p* = .03). Further, at one-year follow-up, the CO monitor repeated assessments revealed a significant decrease of ppm levels in the program group (-0.56, *p* < .001) while the control group showed a significant increase of ppm levels (0.17, *p* < .001). However, one-year follow-up effects on smoking intention did not show a significant difference between the program and control condition (*p* = .30).

**Table 3:** Program Effects Adjusted for Imbalance in Attrition ^a^.

Outcome variables	Program (*n* = 572)			Control (*n* = 393)			Net Effect (Change in Experiment—Change in Control)			
							Net	95% CI		*p* (df = 5)
	Average	*SE*	*p*	Average	*SE*	*p*		Lower	Upper	*p* Value
Smoking intention ^b^	1.60	0.08	.45	1.98	0.27	<.001**	-0.38	-1.36	-0.12	.30
Smoking last month ^c^	0.57	0.18	.97	1.43	0.66	.35	-0.86	-0.19	0.31	.03*
mFTQ Nicotine Dependence ^d^ ^e^	0.59	0.07	<.001**	0.23	0.24	.53	0.36	-0.34	1.12	.04*

**Notes**:

^a^ Adjusted for the specific outcome assessed at baseline, age, gender, and propensity for attrition center is modeled as a random effect.

^b^ 5-Point scale from Definitely not (1) to Very likely (5).

^c^ Number of cigarettes smoked in the last 30 days.

^d^ 5-Point scale from Yes, I already have (1) to No (5).

^e^ Modified Fagerstrom Tolerance Questionnaire score.

* *p* < .05, two-tailed

** *p* < .001, two-tailed

The intent-to treat quit rate comparison was in the right direction, but this comparison failed to approach significance (*p* = .32). Intent-to-treat quit rates were 22.2% for program condition (22 quit and 78 assumed not to quit) and 15.2% for control condition (17 quit and 95 assumed not to quit).

## Discussion

The high rates of teenage smoking in Spain indicate the need to develop effective programs for tobacco prevention and cessation. The Project EX classroom curriculum was designed to produce smoking prevention and cessation effects in the same program. This study tested the one-year effects of the prevention/cessation program among Spanish adolescents.

The Project EX tobacco use cessation program in Russia [11] significantly reduced future smoking expectation, decreased intention to quit smoking, and increased motivation to quit smoking at immediate posttest. The program also resulted in a 7% quit rate at the 6-month follow-up, compared to 0% in the control condition. With the same program in China, four-month follow-up data indicated a 10.5% 30-day quit rate and a 14.3% 7-day quit rate [14], compared to a naturally occurring quit rate of 3%. In the classroom context, Project EX in southern California [17] produced a greater reduction in weekly smoking and intention for smoking in the next 12 months. At 6-and-12 month follow-ups students in the treatment condition experienced a greater reduction in weekly smoking and monthly smoking [2]. In addition, an incremental 8% higher cessation rate was observed at the program schools [15]. Immediate outcomes of Project EX in Spain [9] showed a greater reduction in smoking intention and CO ppm levels. In the present study, the Project EX prevention/cessation program was translated to Spanish and adapted slightly for the Spanish culture. At one-year follow-up, effects on nicotine dependence and last 30-day cigarette smoking show significant differences between program and control condition, in addition to a significant decrease of ppm levels in the program group along with an increase in ppm levels in the control group. These results confirm the hypothesis from the previous study [9]. We expected to find longer-term effects of the program on smoking prevalence. Although smoking intention and cessation effects did not show significant differences between treatment and control groups, we did find an effect across the range of smoking indicating a significant preventive effect.

Project EX as a prevention-cessation program can effect long-term changes. Still, there are several notable limitations. First, recruitment was not easy in Spanish high schools. Readiness to quit smoking is relatively low in Spain, especially among adolescents; changes in the larger social climate may be needed prior to disseminating school-based prevention/cessation programming [15]. Furthermore, there is low motivation among staff to add extra-curricular activities in the schedule. The recruitment rate of schools was 13%. Possibly, we could have been more aggressive in our recruitment strategies. We were not able to offer the school any incentives for participation (e.g., monetary donation to the school). Second, the dropout rate at one-year follow-up questionnaires was 37.6%. However, we did make at least three attempts per school to collect follow-up data, suggesting to us that higher retention rates would be difficult to obtain. Student absentees accounted for most of the non-completion though subject withdrawal from the study accounted for 35% of the non-completion of questionnaires at follow-up. Furthermore, students who failed to return the absentee surveys were contacted by telephone for survey administration; only 41% of persons called were reached and surveyed. The reasons for non-telephone recruitment were no response (52%), the phone number was missing or not operational (36%), and the youth responded with a statement of no interest (12%). It should be mentioned that follow-up was about the same as previous studies [2], with a retention rate at one-year follow-up of 63% in the control group and 66.4% in the control group.

Third, subjects who completed the survey by telephone did not complete the CO readings. However, concordance of results between those who completed the survey and CO measure suggests that youth reported honestly. Finally, Project EX generalization to other Spanish locations has not yet been tested. It would be interesting to replicate this classroom modality in other Spanish cities.

Despite the limitations of this study, the use of a randomized design (albeit only three units per condition) and the large sample size, along with changes observed particularly on level of 30-day smoking and CO levels, suggests promise of Project EX as a cessation/prevention program for Spanish adolescents. Furthermore, there are no well- evaluated cessation intervention programs for adolescent tobacco in Spain, so the results of this study provide evidence about the long-term effectiveness. It is possible that cessation programming should focus on smokers in a school-based clinic setting rather than in the classroom [26].
